# A Rare Differential of Epistaxis

**DOI:** 10.7759/cureus.59981

**Published:** 2024-05-09

**Authors:** Inemesit Akpan, Ijeoma Orabueze, Uwagbae Ibizugbe, Ghida Akhdar, Walter Y Agyeman

**Affiliations:** 1 Internal Medicine, Piedmont Athens Regional Medical Center, Georgia, USA; 2 Internal Medicine, Vassar Brothers Medical Center, Poughkeepsie, USA; 3 Radiology, Cook County Hospital, Chicago, USA

**Keywords:** middle ear tumors, middle ear, acute hemoptysis, glomus tympanicum, epistaxis, paraganglioma

## Abstract

Glomus tympanicum is a type of glomus tumor that affects the middle ear, located at the auricular branch of the vagus nerve. Glomus tumors, in general, are rare, slow-growing tumors and may not require surgery in some patients. It can be challenging to manage due to its hypervascularity, location, and advanced stage of diagnosis. Although glomus tympanicum commonly presents with pulsatile tinnitus and conductive hearing loss, it presented in our patient with large-volume hemoptysis and epistaxis, requiring urgent diagnostic and therapeutic interventions.

We highlight the unique presentation of a 48-year-old female with sudden onset large-volume hemoptysis and epistaxis, leading to the discovery of a hypervascular glomus tympanicum in the right middle ear, identified via MRI. On arrival, her vitals were within normal limits, and a physical examination was pertinent for the obvious ongoing bleeding from her mouth. The examination revealed increased respiratory effort and bilateral crackles. Laboratory values were pertinent for hemoglobin of 11.8 g/dl. Ear^ ^examination revealed a large, vascular-appearing mass filling the right ear. An MRI of the face and neck showed an avidly enhancing 3.7 cm x 1.8 cm x 1.2 cm mass within the right middle ear and mastoid cavity, extending into the external auditory canal and through the eustachian tube into the nasopharynx. The mass was inseparable from the lateral border of the internal auditory canal in the petrous canal. Due to concern for glomus tympanicum, the patient underwent urgent embolization and subsequent tumor resection.

Considering our patient initially presented large-volume hemoptysis, there was concern for alveolar hemorrhage. However, as she had no increased oxygen requirement, there was suspicion of massive epistaxis mistaken for hemoptysis. Due to large volume epistaxis, she underwent urgent embolization as resection could have been challenging due to increased vascularity. It is important to remember that massive epistaxis may not present with blood in the anterior nares, thereby delaying diagnosis and management. Furthermore, probing such tumors should be avoided as it may lead to life-threatening bleeding.

## Introduction

Paragangliomas, also called chemodectomas, are rare, slow-growing tumors with an estimated incidence of up to one to three per 100,000 people annually [1]. They are benign mesenchymal tumors that can arise in different locations, with names referring to the location of the tumor: the 'glomus jugulare tumor' is found at the superior vagal ganglion and the 'glomus typanicum tumor' is located at the auricular branch of the vagus nerve. Complications from glomus tumors include mass effect, compression, and erosion of local structures. Management of such tumors can be challenging due to their hypervascularity, which results in increased bleeding risk; their anatomic location; and their advanced stage at diagnosis, but being that they are slow-growing, they can be managed non-surgically [1].

Glomus tympanicum arises from the paraganglia of the middle ear [2], and given its location, it commonly presents with pulsatile tinnitus and conductive hearing loss, which is unlike our patient’s presentation with sudden-onset hemoptysis and epistaxis [1,3]. This case report highlights the importance of physical examination in finding the large vascular mass, prompting further imaging with an MRI. It also illustrates the diagnostic challenge of atypical presentations of glomus tympanicum. This article was previously presented as a meeting abstract at the Society of Critical Care Medicine in January 2024.

## Case presentation

A 48-year-old female with a history of mastoiditis and subsequent right mastoidectomy in 2016 was complicated by the development of partial right-sided hearing loss that remained stable until about five months prior to her presentation when it suddenly became progressively worse. She presented to the emergency department (ED) with reports of the sudden onset of large-volume hemoptysis and epistaxis with no prior history of such symptoms. She endorsed a rattling breath sound followed by a nosebleed with large quantities of blood with clots. This was accompanied by generalized weakness and mild left-sided, non-radiating pleuritic chest discomfort. She denied the use of antiplatelet therapy, non-steroidal anti-inflammatory drugs (NSAIDs), or any anticoagulants. There was no preceding trauma, upper respiratory symptoms, or prior history of nosebleeds. 

Vitals on arrival showed a blood pressure of 121/74 mmHg, a pulse of 63 bpm, and a respiratory rate of 17 cpm, saturating at 97% on room air. Physical examination revealed obvious ongoing bleeding from the nose and mouth, increased respiratory effort, and bilateral crackles. Laboratory values were pertinent for hemoglobin 11.8 g/dl, hematocrit 34.8%, mean corpuscular volume (MCV) of 88.1 fL, platelet count of 208 x 10*3/ul, international normalized ratio (INR) of 0.97, and partial thromboplastin time (PTT) of 33.7 seconds. The normal coagulation profile placed coagulopathy low on our differential. Chest CT angiography (CTA) showed patchy centrilobular ground glass opacities and mild bronchial wall thickening in the lower lobes, which was concerning for an infectious or inflammatory process. The patient received topical oxymetazoline spray, nasal clamp, and tranexamic acid with improvement in bleeding, after which the anterior nares were assessed, which were void of any friable tissue. There was an initial concern for alveolar hemorrhage, but given the unremarkable chest CT findings and respiratory exam, bronchoscopy was deferred as the bleeding was thought to be pseudo-hemoptysis from aspirated blood secondary to large-volume epistaxis. 

**Figure 1:**
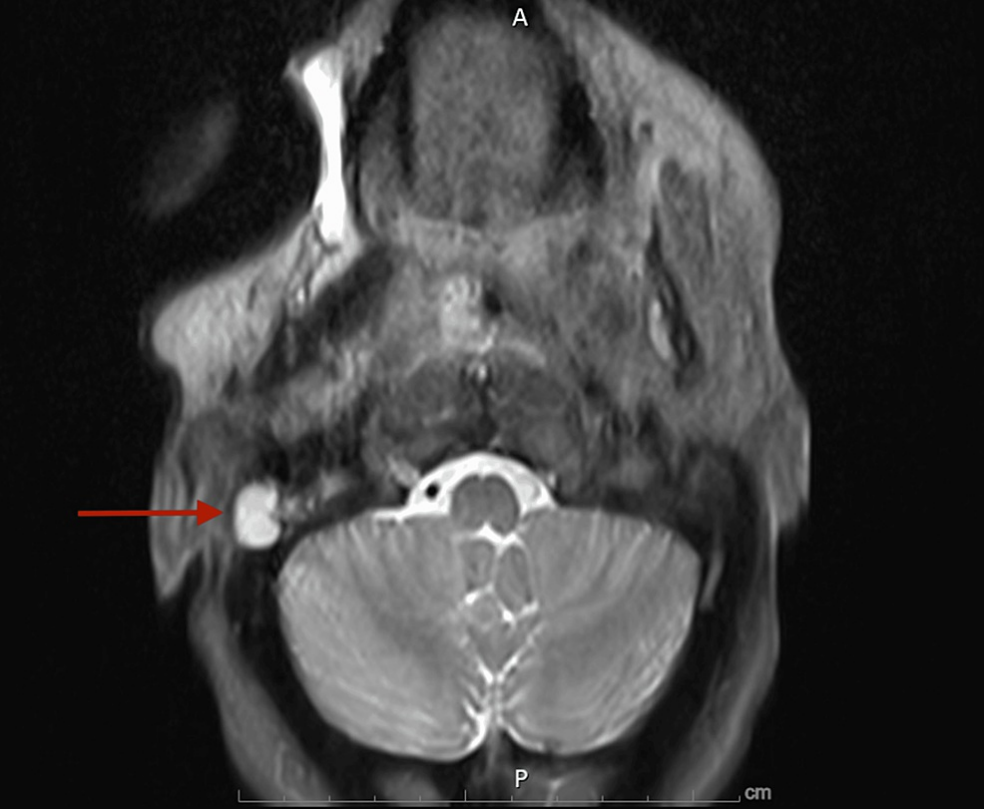
MRI of the orbit, face, and neck with contrast Seen is an avidly enhancing 3.7 cm mass within the right middle ear and mastoid air cells (red arrow) indicating a glomus tympanicum.

Otolaryngology was consulted for further workup of epistaxis, and an ear examination was pertinent for a large, vascular-appearing mass filling the right ear. They recommended an MRI of the orbit, face, and neck to further characterize the vascular-appearing mass, which showed an avidly enhancing 3.7 cm x 1.8 cm x 1.2 cm mass within the right middle ear and mastoid cavity, extending into the external auditory canal and through the eustachian tube into the nasopharynx (Figure 1). The mass was inseparable from the lateral border of the internal auditory canal in the petrous canal. The patient had two more episodes of large-volume hemoptysis that were challenging to control. Due to concern for right-sided paraganglioma, likely glomus tympanicum, based on exam findings and imaging, she was transferred to a quaternary care facility for urgent embolization by neurointerventional radiology to achieve hemostasis. 

The patient was then discharged from the quaternary care facility but about two days later, she had another episode of significant epistaxis of 250 cc measurable blood as well as multiple clots requiring another embolization by neurointerventional radiology. She underwent Fisch subtemporal resection with temporal flap reconstruction about four weeks later. The surgical report found evidence of prior mastoidectomy with the facial nerve exposed at the stylomastoid foramen and a vascular tumor within the mastoid cavity located on and eroding into the lateral semicircular canal and the basal turn of the cochlea. The tumor extended from the middle ear anteromedially along the petrous carotid and temporal lobe dura and nearly to the foramen rotundum. The tumor additionally extended through the cartilaginous eustachian tube. The entirety of the tumor was resected from the middle ear, infratemporal fossa, and eustachian tube. No easy plane of dissection was identified along the petrous carotid artery or temporal lobe dura, and the tumor was subsequently maximally debulked along these structures, resulting in a likely gross total resection. The patient did well post-surgery and was discharged about a week later, with a close follow-up with otolaryngology; she had no further reports of epistaxis. 

## Discussion

The hereditary forms of paragangliomas, which constitute about 20% of paragangliomas, typically have an early onset and can be bilateral [1]. Glomus tympanicum arises from the paraganglia of the middle ear [2]. Only 20% of head and neck paragangliomas have been reported to present as glomus tympanicum, and they commonly present with pulsatile tinnitus and conductive hearing loss [1,3], which is similar to our patient with reports of progressive hearing loss. Rarely do they secrete catecholamines, which can cause systemic symptoms such as hypertension, headaches, flushing, and cardiac arrhythmias [1,4]. 

A physical examination is important in demonstrating a pulsatile, reddish retrotympanic mass [5]. The tympanic membrane can show increased vascularity with an inferiorly based ear mass, giving the appearance of the rising sun, but our patient’s tympanic membrane was not visualized [1,5]. The. mass is most commonly located in the right ear, as seen in our patient, likely due to the more dilated and elevated jugular bulb in that ear. Some patients might present with symptoms of facial nerve palsy based on tumor expansion [3,5]. Incisional biopsy is contraindicated in this tumor due to its highly vascular nature and risk of bleeding [5]. Imaging studies include head CT scans, which can demonstrate bone erosion with a moth-eaten appearance, and MRI with intravenous contrast [1,6]. As this is a slow-growing tumor, some patients can be monitored by imaging alone and may not require surgery [5,7]. In young patients with functional nerve deficits from tumor compression, surgery is recommended [6,8]. Radionuclear medicine, particularly gamma knife radiosurgery, is another treatment option and has a low risk of treatment-related cranial nerve injury [9].

Considering our patient presented with an initial large-volume hemoptysis, there was initial concern for alveolar hemorrhage pending CT imaging. Also, our patient had non-friable nares and no obvious source of bleeding in her nares, suggesting that she was likely having massive epistaxis mistaken for hemoptysis. Due to the location of this tumor and its vascularity, resection can be challenging; thus, preoperative embolization can be helpful [5,10]. Our patient’s recurrent large-volume epistaxis required her to get urgent embolization to help control bleeding.

## Conclusions

Glomus tympanicum can present with significant bleeding in the form of epistaxis or hemoptysis, requiring urgent intervention to improve survival. As physicians, we need to remain aware that massive epistaxis may not present with blood in the anterior nares, thereby delaying diagnosis and management. A multidisciplinary team, especially with otolaryngology, is needed for improved diagnosis and timely management of glomus tympanicum, especially in atypical presentations. Physicians should also remember that probing of such tumors should not be attempted to avoid life-threatening bleeding. More information on various atypical presentations of the glomus tympanicum is needed to better understand the range of presentations and improve timely diagnosis.
